# Polymorphism of Alcohol Metabolizing Gene ADH3 Predisposes to Development of Alcoholic Pancreatitis in North Indian Population

**DOI:** 10.3389/fmolb.2015.00067

**Published:** 2015-12-16

**Authors:** Divya Singh, Tajwar S. Negi, Ghanshyam Upadhyay, Gourdas Choudhuri

**Affiliations:** ^1^Department of Biology, City College of New YorkNew York, NY, USA; ^2^Department of Gastroenterology, Sanjay Gandhi Post Graduate Institute of Medical SciencesLucknow, India; ^3^Department of Gastroenterology and Hepatobiliary Sciences, Fortis Memorial Research InstituteGurgaon, India

**Keywords:** chronic pancreatitis, genetic polymorphism, alcohol dehydrogenase

## Abstract

**Background and aim:** Genetic factors regulating alcohol metabolism could predispose in developing alcoholic pancreatitis (ACP). Studies revealed that alcohol could be metabolized by both ways, oxidative and non-oxidative. The main oxidative pathway includes alcohol dehydrogenase (ADH), aldehyde dehydrogenase (ALDH), and cytochrome P450 enzyme. We investigated the association of polymorphisms in these enzymes with the alcoholic pancreatitis in the north Indian population.

**Method:** Patients with alcoholic pancreatitis (ACP; *n* = 72), tropical calcific pancreatitis (TCP; *n* = 75), alcoholic controls (AC; *n* = 40), and healthy controls (HC; *n* = 100) were included in the study. Blood samples were collected from the subjects in EDTA coated vials. DNA was extracted and genotyping for ADH3, ALDH2, and CYP2E1 was done by PCR-RFLP (polymerase chain reaction—restriction fragment length polymorphism). The products were analyzed by gel electrophoresis.

**Result:** The frequency distribution of ADH3^*^1/^*^1 genotype was significantly higher in ACP group (59.7%) compared with TCP (38.7%), HC (42%), and AC (37.5%) and was found to be associated with increased risk of alcoholic pancreatitis. There was no statistically significant difference between the frequency distribution of ADH3^*^1/^*^1, ADH3^*^1/^*^2, and ADH3^*^2/^*^2 genotypes between TCP and HC or healthy alcoholics. ALDH2 gene was monomorphic in our population, and the frequencies for CYP2E1 intron 6 Dra I polymorphism were comparable in all the four groups.

**Conclusion:** This study shows that carriers of ADH3^*^1/^*^1 individuals consuming alcohol are at higher risk for alcoholic pancreatitis than those with other genotypes such as ADH3^*^1/^*^2 and ADH3^*^2/^*^2.

## Introduction

Alcohol tolerance is highly dependent on the presence or absence of alcohol metabolizing enzymes and the subjects having history of alcohol consumption are more susceptible for chronic pancreatitis onset (Czech and Hartleb, 2003; Cichoz-Lach et al., 2008). Alcohol consumption is suggested to be a major cause of chronic pancreatitis in 70–80% cases in developed countries (Singh and Simsek, 1990; Dzienizewski and Gabryelewicz, 2003; Cichoz-Lach et al., 2008). Genetic factors regulating alcohol metabolism could play a role in developing alcoholic pancreatitis (Vonlaufen et al., 2007). Although these factors are critical in the etiology of chronic pancreatitis, their role in alcoholic pancreatitis is not clearly elucidated.

Alcohol is mainly oxidized by oxidative pathway. The major enzyme of oxidative pathway of alcohol metabolism is alcohol dehydrogenase (ADH)- a dimeric Zn-containing protein with a subunit molecular weight of 40 kDa, and aldehyde dehydrogenase (ALDH; Quertemont, 2004; Edenberg, 2007). ADH is encoded by at least seven gene loci located on the long arm of chromosome 4 (chromosome 4q22) in which ADH2 (ADH1B) and ADH3 (ADH1C) are highly polymorphic (Edenberg, 2000, 2007). ADH3 gene has two alleles ADH3^*^1, ADH3^*^2 encoding subunits γ^1^ (Arginine at position 272 and isoleucine at position 350), and γ^2^ (glutamine at position 272 and a valine at position 350), respectively (Edenberg, 2007). The subunit γ^1^ shows higher ethanol activity than the subunit γ^2^ (Day et al., 1991; Crabb et al., 1993; Edenberg, 2007). In majority of cases the occurrence of these two SNPs have been observed, indicating very high linkage disequilibrium (Edenberg, 2007). The turnover rate of ADH depends on the association of the subunits and the homodimeric enzyme γ1γ1 have substantially higher rate (70%) than γ2γ2 enzyme (Edenberg, 2007). Additionally, ADH3, which encodes a subunit with threonine at position 352 (ADH1C^*^Thr352) has been described in the Native Americans, however, the protein have not been properly investigated (Osier et al., 2002; Edenberg, 2007).

Alcohol is first metabolized to acetaldehyde by ADH and then to acetate by ALDH. Four different isozymes of ALDH; ALDH1, ALDH2, ALDH3, and ALDH4 are found predominantly in the liver and can be differentiated with respect to kinetic properties, localization within the cell, tissue distribution, and electrophoretic mobility (Greenfield and Pietruszko, 1977; Harada et al., 1980; Duley et al., 1985). ALDH2 isozyme is located in mitochondria and is the principal enzyme for acetaldehyde metabolism. ALDH2 is located on the chromosome 12 (chromosome 12q24.2) and is polymorphic with two alleles: ALDH2^*^1 and ALDH2^*^2 with ethnic differences in the polymorphisms and different kinetic properties (Day et al., 1991; Edenberg, 2007).

Moreover, the involvement of Cytochrome P450s (CYPs) has been also implicated in the alcohol metabolism and associated disease progression (Burim et al., 2004; Liu et al., 2007). CYP2E1 is a key microsomal enzyme that metabolizes alcohol in the non-alcohol dehydrogenase pathway (Ingelman-Sundberg et al., 1993; Song, 1996; Liu et al., 2007). Since, CYP2E1 is ethanol inducible and highly polymorphic, it is inevitable to investigate polymorphism in this gene, which may explain the variation in disease causation by alcohol. One important polymorphism of CYP2E1 in the intron 6 region, Dra I polymorphism, has been found to be associated with chronic pancreatitis (Ingelman-Sundberg et al., 1993; Song, 1996).

Polymorphisms in alcohol metabolizing enzyme genes have been found to be associated with alcoholic liver disease (Khan et al., 2009, 2010); however, not much information regarding the role of alcohol metabolizing genes polymorphisms in pancreatitis in the north Indian population is available. Evidence from epidemiological, laboratory, and clinical studies suggest that only about 5–10% alcoholics suffer from clinically recognized CP (Bisceglie and Segal, 1984; Harber et al., 1995; Perri et al., 2003). This variation in disease causation suggests that there could be differences in underlying genetic susceptibility. It can be speculated that polymorphism in these alcohol metabolizing enzyme genes could predispose subjects consuming alcohol to alcoholic pancreatitis. Therefore, the aim of the present study is to investigate the association of ADH3, ALDH2, and CYP2E1 polymorphisms in alcoholic pancreatitis in North Indian population.

## Subjects and methods

### Subjects

All consecutive patients with pancreatitis attending the Pancreatobiliary Clinic at Sanjay Gandhi Post Graduate Institute of Medical Sciences, Lucknow were included in the study as per inclusion criteria. Diagnosis was based on clinical and radiological criteria. Inclusion criteria for tropical pancreatitis were radiological evidence of pancreatic intraductal calculi, no history of alcohol consumption and no family history of Pancreatitis. However, inclusion criteria for alcoholic pancreatitis were history of >80 g of alcohol ingestion for at least 3 years, radiological evidence of pancreatitis, increased serum amylase, and absence of any other cause for pain (Table 1). Alcohol consumption history was taken in a questionnaire. Almost all the patients in both the group (ACP and TCP) had pain at presentation. The pain was epigastric in nature usually radiating to back. Frequency of diabetes in TCP group was 28% (21/75) and in ACP group 18.5% (13/72). The main clinical feature in ACP group was presence of pseudopancreatic cyst. It was present in 41.7% (30/72) of the alcoholic pancreatitis patients. History of alcohol intake was taken in all the patients. None of the patients in TCP group had history of alcohol consumption. In brief, 72 patients with alcoholic pancreatitis, 75 patients with tropical calcific pancreatitis (TCP), 100 healthy controls (HC), and 40 alcoholic controls (AC) free from any gastrointestinal disorder were recruited in the study (Table 2). Cases and controls were from the same ethnic group and were residents of the northern parts of India. The ethics committee of the institution approved the study. A written pre-informed consent was obtained prior to blood collection from the individuals.

**Table 1:** **The inclusion and exclusion criteria for the recruitment of subjects for the study**.

	**Inclusion criteria**	**Exclusion criteria**
ACP	1. History of >80 g of alcohol intake for at least 3 years 2. Radiological evidence of pancreatitis (acute or chronic) 3. Increased serum amylase 4. Absence of any other cause for pain	1. Presence of pancreatic cancer 2. Occasional alcohol intake
TCP	1. Radiological evidence of pancreatic intraductal calcification 2. Non-alcoholic 3. No family history of Pancreatitis	1. Presence of any other etiological factor like alcoholism, gall stones hypercalcaemia etc. 2. Presence of pancreatic cancer

**Table 2:** **Age and Gender distribution**.

**Characteristics**	**ACP**	**TCP**	**HC**	**AC**
*N*	72	75	100	40
Sex (male/female)	All male	40/35	60/40	All male
Age (mean ± SD) in years	38.5 ± 8.3	32.8 ± 11.2	32.05 ± 4.6	35.9 ± 5.3

*ACP, alcoholic chronic pancreatitis; TCP, tropical calcific pancreatitis; HC, healthy control; AC, alcoholic control*.

### Method

#### DNA isolation

Blood sample was collected from each subject in EDTA coated vial. Genomic DNA was extracted by phenol chloroform method previously described (Miller et al., 1998). The purity and integrity of genomic DNA were checked on agarose gel (0.8%) and by calculating the ratio of absorbance at 260/280 nm (1.8–2.0). The DNA content was quantified by absorbance at 260 nm (1.00 optical density was considered equivalent to 50 mg/mL genomic DNA) before PCR amplification. DNA samples were stored at −80°C till further use.

## Genotyping

### Genotyping for ADH3 polymorphism

DNA was amplified with specific oligonucleotide primers. The forward and reverse primers were 5′ GCTTTAAGAGT AAATATTCTGTCCC 3′ and 5′ AATCTACCTC TTTCCGAAGC 3′, respectively. PCR amplification was performed in 25 μl reaction mixture using the standardized PCR condition. The PCR condition included initial denaturation at 95°C for 5 min followed by 35 cycles of denaturation, annealing, and extension at 94°C for 30 s, 55°C for 30 s, and 72°C for 30 s, respectively. Final extension was done at 72°C for 10 min. The 145 bp ADH3 amplicons were digested with SspI restriction enzyme at 37°C for overnight. Genotyping was done by analyzing bands on 12% PAGE. ADH3^1−1^ genotype: two bands (67 and 63 bp), ADH3^1−2^ genotype: three bands (~130, 67, and 63 bp), ADH3^2−2^ genotype: one band (130 bp). A 15 bp cut was also present and not visualized on gel (Groppi et al., 1990).

### Genotyping for ALDH2 polymorphism

ALDH2 genotyping was done by PCR-RFLP. The forward and reverse primers were 5′ CAAATTACAGGGTCAAGGGCT 3′ and 5′ CCACACTCACAGTTTTCTCTT 3′, respectively. PCR was done using standardized PCR condition. The PCR condition included initial denaturation at 95°C for 3 min followed by 35 cycles of denaturation, annealing, and extension at 94°C for 15 s, 59.7°C for 45 s, and 72°C for 30 s, respectively. Final extension was done at 72°C for 10 min. The 134 bp ALDH2 amplicons were digested with MboII restriction enzyme at 37°C for overnight. Genotyping was done by analyzing bands on 12% PAGE. ALDH2^1−1^ genotype: one band (125 bp), ALDH2^1−2^ genotype: two bands (125 and 134 bp), ALDH2^2−2^ genotype: one band (134 bp; Muto et al., 2000).

### Genotyping for CYP2E1 intron 6 (Dra I) polymorphism

Polymorphism in the intron 6 of CYP2E1 was determined by PCR-RFLP method. Enzyme Dra I was used to detect the D and C polymorphism in intron 6. The specific primers were 5′ TCGTCAGTTCCTGAAAGCAGG 3′ and 5′ GAGCTCTGATGGAAGTATCGCA 3′. The PCR condition were initial denaturation at 95°C for 4 min followed by 40 cycles of denaturation, annealing, and extension at 94°C for 1 min, 60.4°C for 45 s, and 72°C for 3 min, respectively. Final extension was done at 72°C for 10 min. The 995 bp amplified product was then digested with Dra I restriction enzyme at 37°C for 15–16 h. Genotyping was done on 3% agarose gel stained with ethidium bromide. DD genotype: three bands (572, 302, and 121 bp), CD genotype four bands (874, 572, 302, and 121 bp), CC genotype: two bands (874 and 121 bp; Kato et al., 1994; Wu et al., 1998).

## Statistical analysis

Genotypic frequencies were calculated and odds ratios (OR) were determined by 2 × 2 contingency table with 95% confidence intervals. Statistical significance for OR was calculated by chi-square test using SPSS Version 12 software. Fisher exact test was used, when expected cell frequency was less than five. Age and sex adjusted OR were calculated to assess the disease risk.

## Results

The mean age and sex distribution are shown in the table (Table 2). Mean age of ACP patients was higher in comparison to TCP patients. Sex distribution in the studied groups was dissimilar as ACP and healthy alcoholics groups consist of males only.

### ADH3 polymorphism

**Figure 1:**
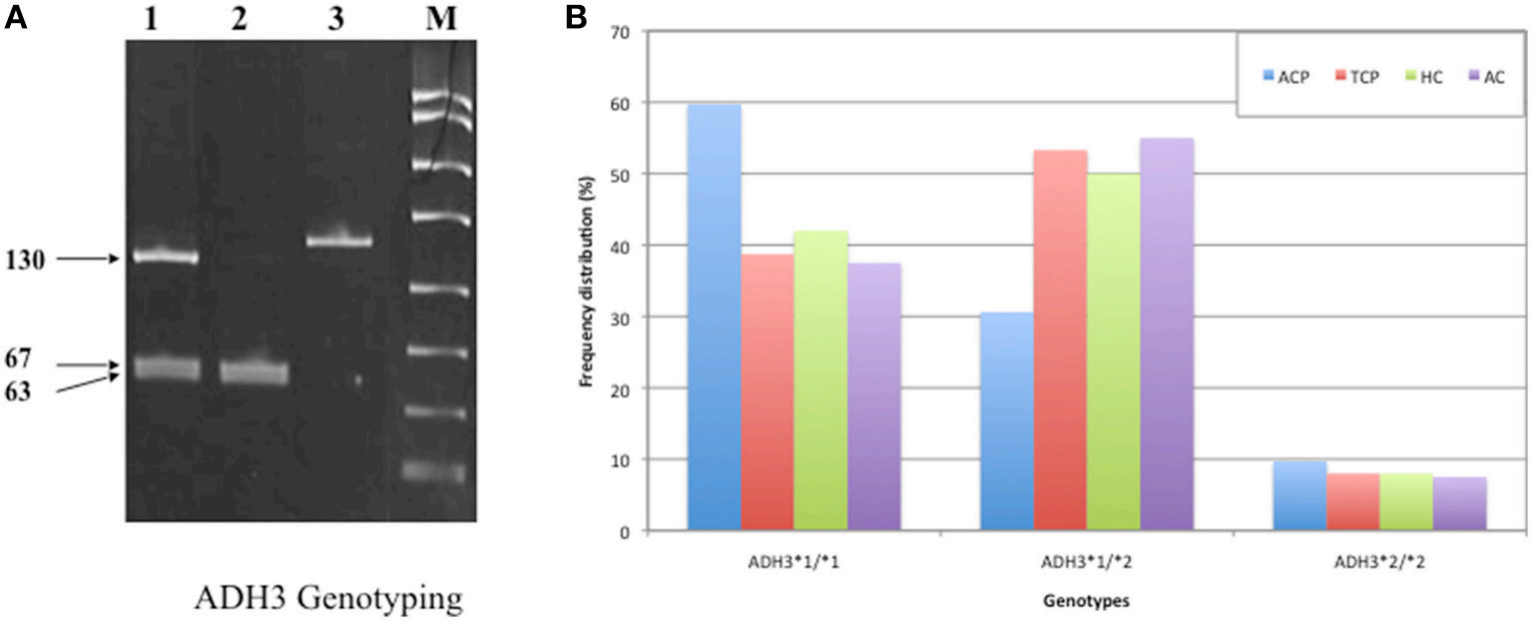
**(A)** Polyacrylamide gel image of PCR amplicons and RFLP by-products of ADH3 gene: Lane1, ADH3^*^1/ADH3^*^2; Lane 2, ADH3^*^1/ADH3^*^1; Lane 3, ADH3^*^2/ADH3^*^2. We observed two bands (67 and 63 bp) for ADH3^1−1^, three bands (~130, 67, and 63 bp) for ADH3^1−2^ and one band (130 bp) for ADH3^2−2^ genotypes. Our result suggest that ADH3^*^1/1 genotypes are more frequent in ACP group in comparison to AC, HC, and TCP and that the carriers of ADH3^*^1/1 genotype are at higher risk for ACP. **(B)** Bar diagram representation of frequency distribution of ADH3 genotypes in ACP, TCP, HC, and AC groups.

Frequency of ADH3^*^1/ADH3^*^1, ADH3^*^1/ADH3^*^2, and ADH3^*^2/ADH3^*^2 genotypes were 59.7 (43/72), 30.6 (22/72), and 9.7% (7/72), respectively in ACP group (Table 3; Figures 1A,B). The prevalence of ADH3^*^1/ADH3^*^1 genotype was higher in ACP group in comparison to TCP, AC, and HC groups. The genotype frequency of ADH3^*^1/ADH3^*^1 was significantly higher in alcoholic pancreatitis in comparison to HC (*p* = 0.01; OR = 2.6; 95% CI = 1.2–5.7) and AC (*p* = 0.02; OR = 2.4; 95% CI = 1.1–5.4). The genotype frequency of ADH3^*^1/ADH3^*^1 was also significantly high in ACP group in comparison to TCP (*p* = 0.01, OR = 2.3; 95% CI = 1.2–4.5) group (Table 4).

**Table 3:** **Summary of frequency distribution of ADH3 genotypes**.

**Genotype**	**ACP (*n* = 72)**	**TCP (*n* = 75)**	**HC (*n* = 100)**	**AC (*n* = 40)**
ADH3^*^1/^*^1	43 (59.7%)	29 (38.7%)	42 (42%)	15 (37.5%)
ADH3^*^1/^*^2	22 (30.6%)	40 (53.3%)	50 (50%)	22 (55%)
ADH3^*^2/^*^2	07 (9.7%)	6 (8%)	08 (8%)	03 (7.5%)

*ACP, alcoholic chronic pancreatitis; HC, healthy control; AC, alcoholic control; TCP, tropical chronic pancreatitis*.

**Table 4:** **Summary of analysis of ADH3 frequency data**.

**Groups**	**ACP vs. TCP**	**ACP vs. HC**	**ACP vs. AC**	**TCP vs. HC**	**TCP vs. AC**	**HC vs. AC**
	***p*-value OR (95% CI)**	***p*-value OR (95% CI)**	***p*-value OR (95% CI)**	***p*-value OR (95% CI)**	***p*-value OR (95% CI)**	***p*-value OR (95% CI)**
ADH3^1−2^ vs. ADH3^1−1^ (ref)	0.06; 0.37 (0.18–0.74)	0.01; 0.43 (0.22–0.88)	0.01; 0.34 (0.15–0.8)	0.6; 1.1 (0.61–2.17)	0.88; 0.94 (0.41–2.11)	0.59; 1.2 (0.56–2.6)
ADH3^2−2^ vs. ADH3^1−1^(ref)	0.69; 0.78 (0.24–2.5)	0.72; 0.85 (0.28–2.5)	0.78; 0.81 (0.18–3.5)	0.88; 1.0 (0.34–3.4)	0.96; 1.0 (0.22–4.7)	0.94; 1.0 (0.24–4.4)
ADH3^1−1^ vs.carriers of ADH3^*2^ (ref)	0.01*; 2.3 (1.2–4.5)	0.01*; 2.6 (1.2–5.7)	0.02*; 2.4 (1.1–5.4)	0.7; 0.89 (0.48–1.6)	0.9; 1.0 (0.47–2.3)	0.67; 0.84 (0.39–1.8)

*ACP, alcoholic chronic pancreatitis; TCP, tropical calcific pancreatitis; HC, healthy control; AC, alcoholic control; OR, odds ratio; CI, confidence interval*.

The prevalence of ADH3^*^1/ADH3^*^1 genotype was 38.7% (29/75) in TCP group. Frequency of ADH3^*^1/ADH3^*^2 and ADH3^*^2/ADH3^*^2 genotype were 53.3 (40/75) and 8% (6/75), respectively in TCP group. ADH3^*^1/ADH3^*^1 genotype frequency in TCP group was comparable with HC and AC groups. Frequencies of both heterozygous ADH3^*^1/ADH3^*^2 genotype and homozygous ADH3^*^2/ADH3^*^2 genotype were statistically similar in TCP, HC, and AC groups (Table 3).

### ALDH2 and CYP2E1 polymorphisms

ALDH2 is an alcohol metabolizing enzyme and polymorphism in this enzyme may affect individual susceptibility to develop the disease (Quertemont, 2004). We performed PCR-RFLP for ALDH2 in 90 patients (ACP: 50, TCP: 40) and 40 each alcoholic (AC) and healthy controls (HC) and found that all were monomorphic having ALDH2^*^1/ALDH2^*^1 homozygote genotype. Additionally, we studied the CYP2E1 intron 6 Dra I polymorphism. In ACP group frequency of DD, CD, and CC genotypes were approximately 85% (61/72), 14% (10/72) and 1.4% (1/72), respectively. Frequency of DD and CD genotypes were 84 (63/75) and 16% (12/75), respectively in TCP group. Frequency distribution of CYP2E1 genotype is statistically similar in all the studied groups (Table 5).

**Table 5:** **CYP2E1 frequency data**.

**Genotype**	**ACP (*n*** = 72**)**	**TCP (*n*** = 75**)**	**HC (*n*** = 90**)**	**AC(*n*** = 40**)**
CC	1 (1.4%)	0	0	0
CD	10 (14%)	12 (16%)	22 (24%)	07 (17.5%)
DD	61 (85%)	63 (84%)	68 (76%)	33 (82.5%)
***P*****-VALUE AND OR (95% CI)**									
	**ACP vs. TCP**	**ACP vs. HC**	**ACP vs. AC**		**TCP vs. HC**	**TCP vs. AC**	**HC vs. AC**
CD	0.9; 1 (0.27–3.7)	0.08; 0.4 (0.14–1.1)	0.5; 0.67 (0.21–3.1)		0.1; 0.5 (0.25–1.2)	0.3; 0.5 (0.1–1.9)	0.3; 0.63 (0.22–1.7)
DD (Ref)	Reference	Reference	Reference		Reference	Reference	Reference

*ACP, alcoholic chronic pancreatitis; TCP, tropical calcific pancreatitis; HC, healthy control; AC, alcoholic control; OR, odds ratio; CI, confidence interval*.

## Discussion

Alcohol misuse is now an established cause of chronic pancreatitis (Verlaan et al., 2004), and therefore the understanding of alcohol metabolism, its regulation, and polymorphisms of the alcohol metabolizing enzymes could be critical for the etiopathogenesis of ACP. There are various determinants of polymorphism, such as, age, sex, location, ethnicity, and race. ADH3, which is involved in the alcohol metabolism, has two alleles ADH3^*^1 and ADH3^*^2 (Edenberg, 2007). Since, ADH3^*^1/ADH3^*^1 genotype encodes for the fast metabolizing enzyme, the subjects carrying this genotype can metabolize ethanol at much faster rate resulting in rapid formation of acetaldehyde (Edenberg, 2007). To retain dynamic homeostasis, the conversion of acetaldehyde to acetate with same pace is essential. Malfunctioning of these proteins due to any mutation may lead the accumulation of acetaldehyde that may account for the disease causation (Majumdar et al., 1986; Nordback et al., 1991; Vonlaufen et al., 2007).

We observed strong association of ADH3^*^1/ADH3^*^1 genotype with alcoholic pancreatitis (Tables 3, 4; ACP vs. HC: *p* = 0.01; OR = 2.6; 95% CI = 1.2–5.7 and ACP vs. AC: *p* = 0.02; OR = 2.4; 95% CI = 1.1–5.4). Studies conducted in past on other populations worldwide have demonstrated varying results—some showing strong association of ADH3 polymorphism with ACP (Day et al., 1991; Dumas et al., 1995), some showing either statistically insignificant or no association of ADH3 polymorphism with ACP (Frenzer et al., 2002; Verlaan et al., 2004). This could be explained as association of ADH3 polymorphism and ACP risk highly depends on the ethnicity therefore could vary from one population to another or one ethnic group to another depending upon the localities they occupy and lifestyle factor.

The ALDH2 gene has two alleles, ALDH2^*^1 and ALDH2^*^2. Since, ALDH2 enzyme is the main form of aldehyde dehydrogenase responsible for acetaldehyde oxidation and polymorphism at its locus has been shown to be associated with chronic pancreatitis (Day et al., 1991), we also investigated ALDH2. Surprisingly, we were unable to detect any polymorphism in ALDH2 and all the subjects were homozygous for ALDH2^*^1/ALDH2^*^1 genotype. It clearly indicates that ALDH2 gene is monomorphic in the north Indian population and it might be possible that our population predominantly carried ALDH2^*^1 allele. Our results were in agreement with the other finding by Bhaskar et al. in six Indian populations, which also showed that all the subjects had common homozygous genotype (ALDH2^*^1/^*^1; Bhaskar et al., 2007). Some other studies conducted previously in Japan and Korea have also shown variable frequency of ALDH2 among different populations (Kimura et al., 2000; Lee et al., 2001).

Furthermore, we also investigated polymorphism of CYP2E1, a key microsomal enzyme induced by ethanol, which metabolizes alcohol in non-alcohol dehydrogenase pathway. We particularly investigated CYP2E1 intron 6D polymorphism, which exhibits two alleles: allele D and allele C. There was no significant difference in the frequency distribution of DD or CD genotype of CYP2E1; however, CC homozygous genotype was less frequent. Genotype frequencies were statistically similar in all the four groups. There was no difference observed in allele frequencies also. The results indicate no association of intron 6 Dra I polymorphism of CYP2E1 with tropical or alcoholic pancreatitis in North Indian population. Our results are in agreement with the previous reports showing no association of CYP2E1 polymorphism with alcoholic cirrhosis (Lee et al., 2001; Vidal et al., 2004). Nevertheless, a study of CYP2E1 intron 6 Dra I polymorphism showed a weak positive association of DD genotype to alcoholic pancreatitis (Verlaan et al., 2004).

In conclusion, the results demonstrate that ADH3 polymorphism may be an important contributor for alcoholic chronic pancreatitis among North Indian population. The polymorphism of ALDH2 and CYP2E1, probably do not have important role in ACP.

### Conflict of interest statement

The authors declare that the research was conducted in the absence of any commercial or financial relationships that could be construed as a potential conflict of interest.
